# Evaluation of a Validated Luminex-Based Multiplex Immunoassay for Measuring Immunoglobulin G Antibodies in Serum to Pneumococcal Capsular Polysaccharides

**DOI:** 10.1128/mSphere.00127-18

**Published:** 2018-08-08

**Authors:** Charles Y. Tan, Fred W. Immermann, Shite Sebastian, Michael W. Pride, Danka Pavliakova, Kelly A. Belanger, Wendy Watson, Daniel A. Scott, Mohinder Sidhu, Kathrin U. Jansen, Peter C. Giardina

**Affiliations:** a Pfizer Inc., Collegeville, Pennsylvania, USA; b Pfizer Inc., Pearl River, New York, USA; c Affinivax, Cambridge, Massachusetts, USA; Parasitology Services

**Keywords:** ELISA, Luminex, *Streptococcus*, immunoassay, pneumonia, vaccine

## Abstract

This article describes the results of a study designed to bridge the World Health Organization (WHO) pneumococcal enzyme-linked immunosorbent assay (ELISA) platform to the validated Luminex-based 13-plex direct immunoassay (dLIA) platform developed by Pfizer, Inc. Both assay platforms quantify serotype-specific serum IgG antibodies (in micrograms per milliliter) against an international reference standard serum. The primary goal of this study was to determine if the dLIA is a suitable replacement for the ELISA to support clinical vaccine studies that include the evaluation of immune responses to serotypes 1, 3, 4, 5, 6A, 6B, 7F, 9V, 14, 18C, 19A, 19F, and 23F. Serum samples were selected from four pivotal 13-valent pneumococcal conjugate vaccine (13vPnC; Prevnar 13) clinical trials on the basis of their serotype-specific IgG concentrations by ELISA. In these studies, subjects were immunized either with 13vPnC or with 7-valent pneumococcal conjugate vaccine (7vPnC; Prevnar). There were 1,528 of 1,574 selected samples with sufficient remaining volume for reanalysis in the dLIA. A comparison of assay results from the dLIA and ELISA platforms showed clear and robust linear quantitative relationships across all 13 serotypes. In addition, lower IgG antibody concentrations in preimmunization samples were measured in the dLIA, thus allowing better differentiation between preimmunization and low-titer postimmunization samples. Overall, the results showed that the established population-level protective threshold IgG concentration, 0.35 µg/ml of serotype-specific serum IgG antibodies, is appropriate for use for data generated using the dLIA platform developed by Pfizer, Inc., for 10 serotypes: serotypes 1, 3, 4, 6A, 7F, 9V, 14, 18C, 19F, and 23F. On the basis of the extensive bridging analyses, however, the use of dLIA cutoff values of 0.23, 0.10, and 0.12 µg/ml is recommended for serotypes 5, 6B, and 19A, respectively. This adjustment will ensure that the consistency of the established population-level protective threshold IgG concentration is maintained when switching from the ELISA to the dLIA platform. The results of this bridging study demonstrate that the 13-plex dLIA platform is a suitable replacement for the WHO reference ELISA platform.

**IMPORTANCE** The pneumococcal enzyme-linked immunosorbent assay (ELISA) measures IgG antibodies in human serum, and it is an important assay that supports licensure of pneumococcal vaccines. The immune correlate of protection, 0.35 µg/ml of IgG antibodies, was determined by the ELISA method. Pfizer has developed a new Luminex-based assay platform to replace the ELISA. These papers describe the important work of (i) validating the Luminex-based assay and (ii) bridging the immune correlate of protection (0.35 µg/ml IgG) to equivalent values reported by the Luminex platform.

## Abstract (precis)

The pneumococcal enzyme-linked immunosorbent assay (ELISA) measures IgG antibodies in human serum, and it is an important assay that supports licensure of pneumococcal vaccines. The immune correlate of protection, 0.35 µg/ml of IgG antibodies, was determined by the ELISA method. Pfizer has developed a new Luminex-based assay platform to replace the ELISA. These papers describe the important work of (i) validating the Luminex-based assay and (ii) bridging the immune correlate of protection (0.35 µg/ml IgG) to equivalent values reported by the Luminex platform.

## INTRODUCTION

Over the past 20 years, the pneumococcal capsular polysaccharide (PnPS) enzyme-linked immunosorbent assay (ELISA) platform has been used to measure levels of serotype-specific serum IgG antibodies to evaluate both established and experimental pneumococcal conjugate vaccine (PCV) formulations. PnPS-specific serum antibodies offer protection against invasive pneumococcal disease (IPD), and in 2003, ELISA data sets from three pivotal efficacy studies in infants were used to establish a population-level protective serum IgG threshold concentration of 0.35 µg/ml (1–4). Vaccine efficacy was shown to correlate with this threshold value in infant populations immunized against 7vPnC serotypes 4, 6B, 9V, 14, 18C, 19F, and 23F (5–7).

The World Health Organization (WHO) Expert Committee on Biological Standardization endorsed this threshold IgG concentration for infant studies in a comprehensive guidance document for PCV manufacturers (7–9). However, this cutoff value is not applicable to adult studies. Serum samples from unimmunized adults often contain cross-reactive, low-avidity IgG antibodies and anti-cell wall polysaccharide (anti-CWPS)-specific IgG antibodies in significant amounts that are detected by the ELISA but do not confer protection against IPD (10). Therefore, functional antibody titers, measured by the opsonophagocytic activity (OPA) assay, were deemed more relevant for the evaluation of vaccine performance in adult populations than IgG measurements (11). The relationship between serum IgG concentration and PCV efficacy in infants continues to be important for evaluating the performance of PCV formulations, such as 13-valent pneumococcal conjugate vaccine (13vPnC; Prevnar 13), which contains six additional PnPS conjugates, serotypes 1, 3, 5, 6A, 7F, and 19A, as well as the original seven 7vPnC serotypes (12–15).

An examination of the percentage of vaccine responders reaching IgG concentrations of ≥0.35 µg/ml is historically based on the WHO reference ELISA platform, and the WHO Expert Committee has provided guidance on acceptable bridging strategies for manufacturers that use an alternative assay platform for clinical evaluations (7). According to this guidance, alternative methods used to evaluate protection against IPD by assessing IgG concentrations in serum from vaccinated infants should be adequately bridged to the WHO ELISA by two major criteria (7). First, the new method should have well-justified threshold values that correspond to the established 0.35 µg/ml ELISA-based benchmark; second, a suitable regression procedure, such as Deming regression, should be used to analyze the data (7, 16). This requirement provides a direct link back to the ELISA platform and the original 0.35 µg/ml threshold value for 7vPnC serotypes. Furthermore, for each serotype, 85% (later revised to 75%) of the WHO reference ELISA results should be within 40% of the results generated by the alternative assay platform using a panel of infant samples obtained from 7vPnC clinical studies or another approved pneumococcal conjugate vaccine with expanded valency, should 7vPnC be no longer available (7, 17). The second criterion provides a way to evaluate assay equivalence (i.e., interlaboratory evaluation of a single assay platform) but offers less value in the platform bridging process because it does not allow for improvements in factors such as specificity and dynamic range in new assay platforms.

Pfizer has developed a Luminex-based direct immunoassay (dLIA) platform to replace the WHO reference ELISA platform. The dLIA platform has been validated for the 13 PnPS serotypes found in 13vPnC. Both platforms quantify serotype-specific serum IgG antibodies against an international reference standard with known IgG concentrations (18). However, unlike the ELISA procedure, which relies on passive adsorption of PnPS to polystyrene assay plates, the dLIA procedure uses a standard chemical process to couple poly-l-lysine (PLL)-conjugated PnPS to fluorescent Luminex microspheres. Each PnPS-PLL conjugate is coupled to spectrally distinct microspheres, which are pooled for the multiplex assay procedure. The targeted coupling of PnPS-PLL-conjugated antigens to microspheres reduces the presence of CWPS on the solid support, which improves the overall specificity of the assay. Furthermore, the dLIA platform uses less serum than the ELISA, which is important for infant studies, where serum volumes are limited.

This bridging study was designed to evaluate the performance of the multiplex dLIA platform and compare it to the performance of the WHO reference ELISA. Furthermore, the study was performed to provide the data to support the use of the dLIA platform as a suitable replacement for the ELISA to support clinical PCV studies. Serum samples were chosen from four pivotal 13vPnC clinical trials in the United States, Germany, and Japan in which subjects received the 7vPnC vaccine or the 13vPnC vaccine (12, 14, 15, 19). Samples with sufficient remaining volume to be reanalyzed in the dLIA were selected from the post-primary-immunization series and postbooster time points. This article provides an assessment of the performance of the multiplex dLIA platform against the WHO reference ELISA and establishes well-justified alternative threshold IgG concentrations for the dLIA platform that correspond to the 0.35 µg/ml ELISA benchmark.

## RESULTS

### Deming regression analysis of the linear relationship between ELISA and dLIA platforms.

The quantitative relationships between the WHO reference ELISA platform and the multiplex dLIA platform were evaluated by Deming regression of log-transformed IgG values for each serotype. In a limited number of cases, there was insufficient residual serum volume to generate a full set of 13 dLIA results. In those cases, the ELISA result did not have a paired dLIA result and was not used in the regression analysis. The total proportion of missing data was less than 1.31% for all serotypes. Data-derived dLIA threshold values corresponding to the WHO ELISA 0.35 µg/ml benchmark were computed for each serotype from the corresponding linear model. Table 1 shows the data sets used for the Deming regression analysis. The primary data set is comprised of all postimmunization samples, including post-infant-immunization series (study visit 4) and post-toddler dose (booster; study visit 6 or 8), while the smaller secondary data set includes only samples collected at study visit 4 after the infant immunization series. The additional data sets listed in Table 1 are comprised of one or more serum panels within a given study. They are subsets of the larger primary and secondary data sets and are used to further investigate assay performance in various populations. Table 2 and Table 3 summarize the results by serotype for the primary and secondary data sets, respectively, including the sample size, the linear relationship established by Deming regression based on the primary data set, and the derived dLIA threshold value based on this linear relationship for each serotype. The 95% confidence interval (CI) of the slope of the Deming regression curve is an indication of the strength of the linear relationship; the tighter the confidence interval, the stronger the linear relationship.

**TABLE 1:** Data sets used for Deming regression analysis

Data set	Trial(s)	Visit(s)	Serum panels included
Primary	All trials	All post^b^	A1, A2, A3^a^, A4^a^, B1, B2, B3^a^, B4^a^, C1, C2, C3^a^, C4^a^, D2, D4^a^, E1, E2
			
Secondary	All trials	4	A1, A3^a^, B1, B3^a^, C1, C3^a^, D2, D4^a^
			
Additional			
Set 1	6096A1-006	4	A1, A3^a^
Set 2	6096A1-006	4, 6	A1, A2, A3^a^, A4^a^
Set 3	6096A1-006	4, 5, 6	A1, A2, A3^a^, A4^a^, E1
Set 4	6096A1-3005	4	B1, B3^a^
Set 5	6096A1-3005	4, 6	B1, B2, B3^a^, B4^a^
Set 6	6096A1-3024	4	C1, C3^a^
Set 7	6096A1-3024	4, 8	C1, C2, C3^a^, C4^a^
Set 8	6096A1-3024	4, 7, 8	C1, C2, C3^a^, C4^a^, E2
Set 9	6096A1-003	4	D2, D4^a^

a Data sets used for serotypes 4, 6B, 9V, 14, 18C, 19F, and 23F only, as subjects received 7vPnC.

b All post, all postimmunization samples.

**TABLE 2:** Relationships between ELISA and dLIA serum IgG concentrations and calculated dLIA threshold based on the primary data set

Serotype	*n^a^*	Deming regression	(95% CI^b^ for slope), width	dLIA threshold/ IgG concn (µg/ml)
1	783	Log_10_(dLIA) = 1.04 × log_10_(ELISA) − 0.07	(1.018, 1.061), 0.043	0.29
3	783	Log_10_(dLIA) = 1.08 × log_10_(ELISA) + 0.16	(1.039, 1.133), 0.094	0.46
4	1,337	Log_10_(dLIA) = 1.16 × log_10_(ELISA) + 0.09	(1.133, 1.193), 0.060	0.37
5	783	Log_10_(dLIA) = 1.30 × log_10_(ELISA) − 0.15	(1.250, 1.348), 0.098	0.18
6A	783	Log_10_(dLIA) = 1.32 × log_10_(ELISA) + 0.11	(1.280, 1.356), 0.076	0.32
6B	1,337	Log_10_(dLIA) = 1.38 × log_10_(ELISA) − 0.37	(1.359, 1.411), 0.052	0.10
7F	783	Log_10_(dLIA) = 1.12 × log_10_(ELISA) + 0.08	(1.079, 1.162), 0.083	0.37
9V	1,337	Log_10_(dLIA) = 1.28 × log_10_(ELISA) + 0.16	(1.235, 1.330), 0.095	0.38
14	1,337	Log_10_(dLIA) = 1.10 × log_10_(ELISA) − 0.03	(1.077, 1.127), 0.050	0.29
18C	1,337	Log_10_(dLIA) = 1.14 × log_10_(ELISA) + 0.21	(1.106, 1.166), 0.060	0.49
19A	783	Log_10_(dLIA) = 1.30 × log_10_(ELISA) − 0.34	(1.262, 1.344), 0.082	0.12
19F	1,337	Log_10_(dLIA) = 1.06 × log_10_(ELISA) + 0.07	(1.041, 1.077), 0.036	0.39
23F	1,334	Log_10_(dLIA) = 1.27 × log_10_(ELISA) + 0.18	(1.239, 1.294), 0.055	0.40

a *n*, number of samples in the primary data sets.

b CI, confidence interval.

**TABLE 3:** Relationships between ELISA and dLIA serum IgG concentrations and calculated dLIA threshold based on the secondary data sets by serotype

Serotype	*n^a^*	Deming regression	(95% CI^b^ for slope), width	dLIA threshold/ IgG concn (µg/ml)
1	298	Log_10_(dLIA) = 1.06 × log_10_(ELISA) − 0.08	(1.021, 1.091), 0.070	0.27
3	298	Log_10_(dLIA) = 1.10 × log_10_(ELISA) + 0.05	(1.036, 1.171), 0.135	0.35
4	603	Log_10_(dLIA) = 1.21 × log_10_(ELISA) − 0.05	(1.160, 1.258), 0.098	0.25
5	298	Log_10_(dLIA) = 1.27 × log_10_(ELISA) − 0.05	(1.208, 1.344), 0.136	0.23
6A	298	Log_10_(dLIA) = 1.49 × log_10_(ELISA) + 0.08	(1.418, 1.562), 0.144	0.25
6B	603	Log_10_(dLIA) = 1.35 × log_10_(ELISA) − 0.38	(1.315, 1.391), 0.076	0.10
7F	298	Log_10_(dLIA) = 1.19 × log_10_(ELISA) − 0.03	(1.112, 1.280), 0.168	0.27
9V	603	Log_10_(dLIA) = 1.28 × log_10_(ELISA) + 0.06	(1.204, 1.361), 0.157	0.30
14	603	Log_10_(dLIA) = 1.08 × log_10_(ELISA) − 0.11	(1.051, 1.121), 0.070	0.25
18C	603	Log_10_(dLIA) = 1.21 × log_10_(ELISA) + 0.08	(1.157, 1.265), 0.108	0.34
19A	298	Log_10_(dLIA) = 1.33 × log_10_(ELISA) − 0.27	(1.270, 1.400), 0.130	0.13
19F	603	Log_10_(dLIA) = 1.07 × log_10_(ELISA) + 0.08	(1.042, 1.098), 0.056	0.39
23F	603	Log_10_(dLIA) = 1.25 × log_10_(ELISA) + 0.10	(1.213, 1.294), 0.081	0.34

a *N*, number of samples of the secondary data sets.

b CI, confidence interval.

The CI values for the slopes of the Deming regression curves were generally within 2% to 4% of the corresponding slope estimates for serotypes 1, 4, 6A, 6B, 7F, 9V, 14, 18C, 19A, 19F, and 23F, which indicates a strong linear relationship between the ELISA and dLIA IgG results. The CI of the slope for serotype 3 was within 5% of the estimated slope for the primary data set and was within 6.5% for the secondary data set. For serotype 5, the CI of the slope was within 4% of the estimated slope for the primary data set and within 6% for the secondary data set. Note that the CI based on the secondary data set is wider than that based on the primary data set because the primary data set has a larger sample size as well as a larger spread of data.

**FIG 1:**
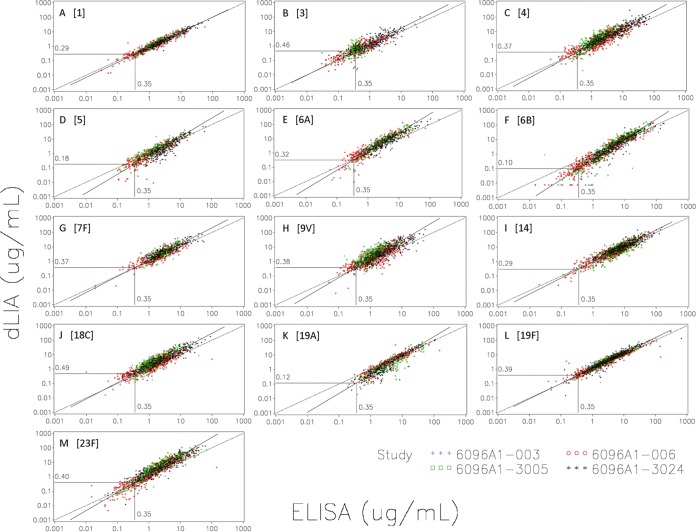
Primary data set scatter plots of the dLIA results on the *y* axis versus the ELISA results on the *x* axis. (A) PnPS 1. (B) PnPS 3. (C) PnPS 4. (D) PnPS 5. (E) PnPs 6A. (F) PnPS 6B. (G) PnPS 7F. (H) PnPS 9V. (I) PnPS 14. (J) PnPS 18C. (K) PnPS 19A. (L) PnPS 19F. (M) PnPS 23F. The dashed reference line represents the *y* = *x* concordance line corresponding to a theoretical perfect match between the two assay platforms. The solid line represents the fitted Deming regression curve based on the primary data set. The vertical line ascending from the *x* axis to the fitted regression curve represents the 0.35 µg/ml IgG threshold by ELISA. The horizontal line extending from this point on the fitted regression curve to the *y* axis represents the corresponding value on the *y* axis for the dLIA platform.

**FIG 2:**
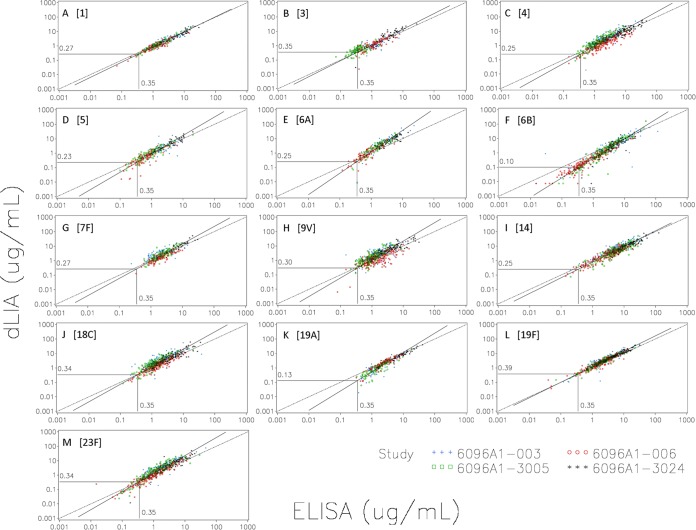
Secondary data set scatter plots of the dLIA results on the *y* axis versus the ELISA results on the *x* axis. (A) PnPS 1. (B) PnPS 3. (C) PnPS 4. (D) PnPS 5. (E) PnPs 6A. (F) PnPS 6B. (G) PnPS 7F. (H) PnPS 9V. (I) PnPS 14. (J) PnPS 18C. (K) PnPS 19A. (L) PnPS 19F. (M) PnPS 23F. The dashed reference line represents the *y* = *x* concordance line corresponding to a theoretical perfect match between the two assay platforms. The solid line represents the fitted Deming regression curve based on the primary data set. The vertical line ascending from the *x* axis to the fitted regression curve represents the 0.35 µg/ml IgG threshold by ELISA. The horizontal line extending from this point on the fitted regression curve to the *y* axis represents the corresponding value on the *y* axis for the dLIA platform.

Figure 1 and Fig. 2 show scatter plots of the dLIA results on the *y* axis versus the ELISA results on the *x* axis based on the primary and secondary data sets, respectively. The dashed reference line represents the *y* = *x* concordance line corresponding to a theoretical perfect match between the two assay platforms. The solid line represents the fitted Deming regression curve based on the primary data set. The vertical line ascending from the *x* axis to the fitted regression curve represents 0.35 µg/ml on the *x* axis (ELISA), and the horizontal line extending from this point on the fitted regression curve to the *y* axis represents the corresponding IgG concentration on the *y* axis (dLIA). For example, a value of 0.35 µg/ml on the *x* axis (ELISA) corresponds to a value of 0.29 µg/ml on the *y* axis (dLIA) for serotype 1 on the primary data set (see Fig. 1A). These scatter plots provide a visual representation of the derived dLIA threshold values that are listed in Tables 2 and 3 for the primary and secondary data sets, respectively.

For serotypes 1, 4, 6A, 7F, 9V, 14, 18C, 19F, and 23F, the estimated dLIA threshold values based on Deming regression of the primary data set were shown to be within the range of 0.29 to 0.49 µg/ml (Table 2). However, for serotypes 5, 6B, and 19A, the estimated threshold values were shown to be 0.18, 0.10, and 0.12, respectively. For serotype 3, the estimate was shown to be 0.46 µg/ml based on the larger primary data set and 0.35 µg/ml based on the secondary data set, which consists only of samples taken after the infant immunization series (Table 3).

### Robustness of derived threshold values.

The data subsets listed in Table 1 were used to evaluate the robustness of each of the estimated dLIA threshold values based on regression analysis of the primary data set (data not shown). The estimated dLIA threshold values based on the primary data set for serotypes 1, 4, 6A, 7F, 9V, 14, 18C, 19F, and 23F were shown to be robust and to be close to 0.35 µg/ml. However, for serotypes 5, 6B, and 19A, the threshold values derived on the basis of the data subsets were all lower than 0.35 µg/ml, and these results were consistent with the dLIA threshold values based on the primary data set, which were shown to be 0.18, 0.10, and 0.12, respectively. Also, for these three serotypes, the dLIA platform measured lower serum IgG antibody concentrations in immunized subjects at the low end of the distribution than the ELISA. This result is likely due to the improved specificity of the dLIA method over the ELISA. The specificity of the dLIA platform is described in another paper (18).

The threshold values derived on the basis of the primary and secondary data sets for serotype 3 were shown to be 0.46 and 0.35 µg/ml, respectively. However, note that the dLIA threshold values for serotype 3, derived on the basis of the data sets from individual clinical studies, fell within a relatively wide range of 0.12 to 0.54 µg/ml but were generally higher than 0.35 µg/ml.

### Analysis of immunized and unimmunized populations.

The second objective of this study was to examine the ability of the dLIA platform to differentiate immunized from unimmunized populations compared to the WHO reference ELISA. The early phase 1/2 clinical trial, 6096A1-003 (United States), was selected for this analysis since it included serum samples collected prior to the first immunization (12). Serum panels D1 to D4 were used for the analysis (Table 4). Serum panels D1 (unimmunized) and D2 (immunized) contain paired samples collected prior to the first dose of 13vPnC and following the third dose of the infant series, respectively. Likewise, serum panels D3 (unimmunized) and D4 (immunized) contain samples collected prior to the first dose of 7vPnC and following the infant series.

**TABLE 4:** List of serum panels and the number of serum samples per panel

Panel	Country	Clinical trial	Vaccine treatment	Study visit	Study time point	Planned no. of samples	Actual no. of samples^a^
A1	Germany	6096A1-006	13vPnC	4	1 mo after infant series	100	96
A2	Germany	6096A1-006	13vPnC	6	1 mo after toddler dose	100	96
A3	Germany	6096A1-006	7vPnC	4	1 mo after infant series	100	100
A4	Germany	6096A1-006	7vPnC	6	1 mo after toddler dose	100	98
B1	United States	6096A1-3005	13vPnC	4	1 mo after infant series	100	100
B2	United States	6096A1-3005	13vPnC	6	1 mo after toddler dose	100	100
B3	United States	6096A1-3005	7vPnC	4	1 mo after infant series	100	100
B4	United States	6096A1-3005	7vPnC	6	1 mo after toddler dose	100	100
C1	Japan	6096A1-3024	13vPnC	4	1 mo after infant series	45	45
C2	Japan	6096A1-3024	13vPnC	8	1 mo after toddler dose	43	43
C3	Japan	6096A1-3024	7vPnC	4	1 mo after infant series	37	37
C4	Japan	6096A1-3024	7vPnC	8	1 mo after toddler dose	51	51
D1	United States	6096A1-003	13vPnC	1	Before the 1st dose	91	91
D2	United States	6096A1-003	13vPnC	4	1 mo after infant series	61	57
D3	United States	6096A1-003	7vPnC	1	Before the 1st dose	100	100
D4	United States	6096A1-003	7vPnC	4	1 mo after infant series	73	68
E1	Germany	6096A1-006	13vPnC	5	Before toddler dose	102	75
E2	Japan	6096A1-3024	13vPnC	7	Before toddler dose	171	171

a Actual number of selected samples based on ELISA with sufficient residual volume to be tested in the dLIA.

**FIG 3:**
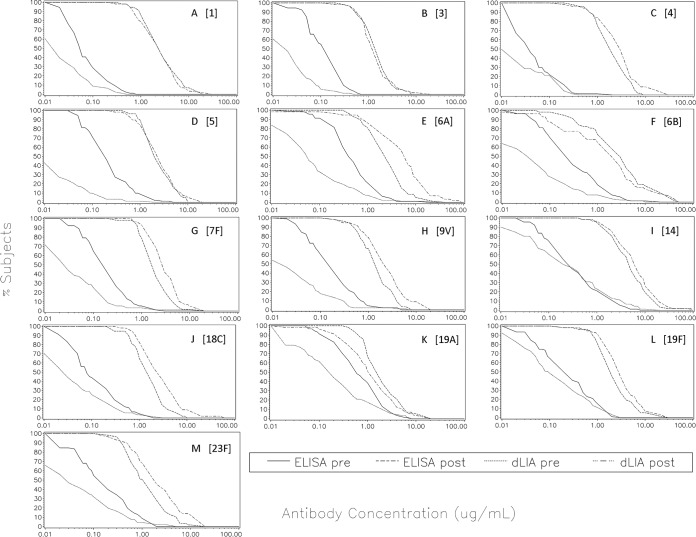
13vPnC reverse cumulative distribution curve plots pre- and postimmunization. Superimposed reverse cumulative distribution curves of the ELISA and dLIA IgG results, pre- and postimmunization, are shown for each serotype in the 13vPnC vaccine from serum panels D1 and D2 from the 13vPnC arm of the clinical study. *x* axis: IgG concentration in micrograms per milliliter. *y* axis: percentage of subjects. (A) PnPS 1. (B) PnPS 3. (C) PnPS 4. (D) PnPS 5. (E) PnPs 6A. (F) PnPS 6B. (G) PnPS 7F. (H) PnPS 9V. (I) PnPS 14. (J) PnPS 18C. (K) PnPS 19A. (L) PnPS 19F. (M) PnPS 23F.

**FIG 4:**
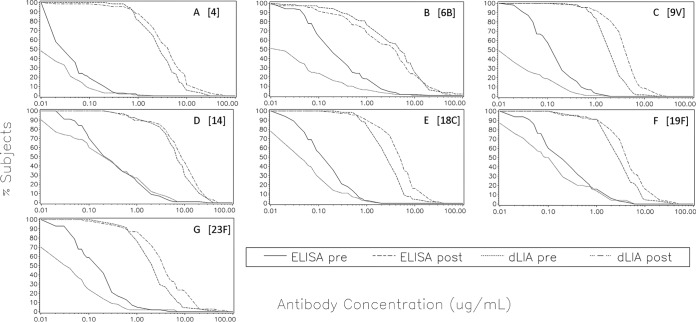
7vPnC reverse cumulative distribution curve plots pre- and postimmunization. Superimposed reverse cumulative distribution curves of the ELISA and dLIA IgG results, pre- and postimmunization, are shown for each serotype in the 7vPnC vaccine from serum panels D3 and D4 from the 7vPnC arm of the clinical study. *x* axis: IgG concentration in micrograms per milliliter. *y* axis: percentage of subjects. (A) PnPS 4. (B) PnPS 6B. (C) PnPS 9V. (D) PnPS 14. (E) PnPS 18C. (F) PnPS 19F. (G) PnPS 23F.

Figure 3 shows the superimposed reverse cumulative distribution curve (RCDC) plots of the ELISA and dLIA results for each serotype in 13vPnC from serum panels D1 and D2 from the 13vPnC arm of the clinical study. These RCDC plots were used to qualitatively visualize the separation between the immunized and unimmunized study populations. Each panel in the figure shows RCDC plots for 1 of the 13 serotypes in the 13vPnC formulation. Likewise, Fig. 4 shows superimposed RCDC plots for serum panels D3 and D4 from the 7vPnC arm of the study.

Both assay platforms showed a separation between the immunized and unimmunized populations, as expected. However, the results show a further separation between the dLIA and ELISA platforms for the unimmunized population, and this observation is consistent for all 13 serotypes and both arms of the study. The RCDC plots of the dLIA results are shifted to the left of the RCDC plots derived from the ELISA platform for the preimmunization population, which indicates that the dLIA platform measures lower levels of serum IgG antibodies in the unimmunized subjects. These results suggest that the dLIA platform is more specific than the ELISA and is therefore better able to differentiate the immunized and unimmunized populations.

### Analysis of the proportions of vaccine responders.

Pneumococcal vaccine response rates are expressed as the percentage of individuals in a population who achieve a serotype-specific serum IgG concentration at or above the established benchmark value of 0.35 µg/ml by ELISA. The third objective of this study was to compare the proportion of vaccine responders indicated by ELISA to the theoretical response rate indicated by the dLIA platform using either the 0.35-µg/ml cutoff value or the derived dLIA cutoff value, as shown in Table 2 (primary data set) or in Table 3 (secondary data set). The theoretical dLIA responder rates were compared to the ELISA responder rates for the individual serum panels using McNemar’s test for paired data (16, 20). To summarize the results for the individual serum panels, the per-serum-panel McNemar *P* values were combined for each serotype using Fisher’s method for combining *P* values (21).

Table 5 lists the combined *P* values for 13 serotypes. In this table, there are 14 serum panels for serotypes 4, 6B, 9V, 14, 18C, 19F, and 23F, which represent subjects immunized with either 7vPnC or 13vPnC. The other serotypes, 1, 3, 5, 6A, 7F, and 19A, include 7 serum panels, which represent subjects immunized with 13vPnC. Serotype 5 also includes an analysis using 4 serum panels representing only serum samples collected after the primary infant dosing series.

**TABLE 5:**
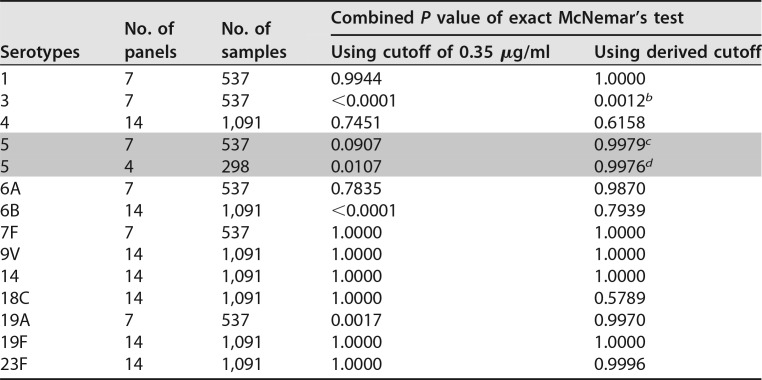
Comparison results of McNemar’s test for 0.35 µg/ml dLIA cutoff values versus derived dLIA cutoff values using the primary or secondary data sets^a^

a Gray-shaded data signify that the evaluation of serotype 5 also includes an analysis using four serum panels representing only serum samples collected after the primary infant dosing series.

^*b*^Refer to Table 6 for an evaluation of serotype 3 results by serum panel.

^*c*^Results are based on the primary data set with a derived cutoff value of 0.18 µg/ml.

^*d*^Results are based on the secondary data set with a derived cutoff value of 0.23 µg/ml.

When 0.35 µg/ml was used as the dLIA cutoff value, the combined *P* values were all substantially higher than 0.25 for serotypes 1, 4, 6A, 7F, 9V, 14, 18C, 19F, and 23F. These results indicate that the vaccine responder rates for the two assay platforms, using the 0.35 µg/ml benchmark, were comparable for these nine serotypes. The 0.35-µg/ml cutoff value and the derived dLIA cutoff values performed similarly in terms of matching the proportions of responders between the two assay platforms. However, for serotypes 5, 6B, and 19A, the combined *P* values were lower than 0.05 when the 0.35-µg/ml cutoff value was used for the dLIA, and therefore, this cutoff value did not perform similarly in determining the proportion of responders. The theoretical cutoff values of 0.23 µg/ml for serotype 5, 0.10 µg/ml for serotype 6B, and 0.12 µg/ml for serotype 19A generated *P* values that were well above the high alpha threshold value of 0.25. These dLIA cutoff values bridged well to the established, ELISA-derived value of 0.35 µg/ml in matching the proportions of vaccine responders.

The appropriate dLIA cutoff value for serotype 3 was not initially obvious from the analysis because there were some serum-panel-to-serum-panel differences (Table 6). The dLIA cutoff values derived from the primary data set (all data) and secondary data set (post-primary immunization) were 0.46 µg/ml and 0.35 µg/ml, respectively. Neither of these cutoff values was satisfactory for all four trials and seven serum panels shown in Table 6. The two assay platforms were in agreement when a cutoff value of 0.35 µg/ml was used for the three serum panels from studies 6096A1-3024 (Japan) and 6096A1-003 (United States), but not for the four serum panels from studies 6096A1-006 (Germany) and 6096A1-3005 (United States). This outcome is an artifact of sample availability in serum panel B2 and the difficulty in selecting a serum panel that conforms to the underlying population distribution for all 13 serotypes. Assay results were clustered near 0.35 µg/ml and did not span the assay range, especially for serum panel B2, which is composed of samples from the post-toddler-boost visit of clinical study 6096A1-3005. Therefore, a cutoff value of 0.35 µg/ml is the recommended dLIA cutoff value for serotype 3 because it works well for the serum panels where the underlying study population is adequately represented.

**TABLE 6:** Comparison of percent responder data for serotype 3 by serum panel

Panel	Clinical study	Time point	No. of samples	ELISA	dLIA (0.35 µg/ml)		dLIA (0.46 µg/ml)	
				%resp^a^	%resp	*P* value	%resp	*P* value
A1	6096A1-006	Post-infant series	96	98.96	91.67	0.0156	86.46	0.0005
A2	6096A1-006	Postboost	96	88.54	95.83	0.0391	93.75	0.1797
B1	6096A1-3005	Post-infant series	100	58.00	79.00	<0.0001	58.00	1.0000
B2	6096A1-3005	Postboost	100	58.00	92.00	<0.0001	81.00	0.0004
C1	6096A1-3024	Post-infant series	45	97.78	97.78	1.0000	97.78	1.0000
C2	6096A1-3024	Postboost	43	100.00	100.00	1.0000	100.00	1.0000
D1	6096A1-003	Post-infant series	57	98.25	96.49	1.0000	94.74	0.5000

a %resp, percent responders.

## DISCUSSION

Pfizer licensed Prevnar 13 for use in infants and toddlers based on serum IgG results from clinical studies measured by the WHO reference ELISA. To improve assay specificity, CWPS and CWPS2 were used in serum dilution buffer to absorb antibodies that might otherwise bind to trace amounts of these antigens on the coated assay plates and thus artificially increase the serotype-specific IgG estimates for the test samples. However, since the published IgG assignments for the 89SF reference standard were generated under single-absorbent assay conditions, only CWPS is used to prepare the reference serum dilutions for the WHO reference ELISA (5, 7). Although this approach led to lower serotype-specific IgG estimates for clinical samples, it was necessary to maintain the link to the population-level efficacy threshold, 0.35 µg/ml of IgG, recommended by the WHO Expert Committee (5–7). A new reference standard serum, 007sp, was recently developed by the U.S. FDA to replace the 89SF material, and a carefully planned multicenter study was conducted to bridge the 89SF IgG estimates under single-absorbent assay conditions to 007sp using both CWPS and CSPW2 (22). Again, this approach was necessary to maintain the link to the 0.35 µg/ml IgG threshold and the original efficacy studies, and the WHO Expert Committee has recommended bridging studies to address any changes to the WHO reference ELISA or other suitable assay platform (7).

Recent advances in multiplex bead-based technologies have encouraged researchers to develop assays to replace the ELISA platform. There are several advantages to using a multiplex assay approach over the WHO reference ELISA for testing infant clinical samples. Luminex-based assay procedures require less serum to generate results for all 13 serotypes. For example, the ELISA requires as much as 70 μl of serum and 13 coated assay plates to test a given sample against 13 pneumococcal serotypes. The same number of tests can be achieved by the multiplex dLIA platform with 5 to 10 μl of serum, depending on the size of the sample dilution volume. Also, the multiplex approach requires less time and generates far less laboratory waste. The 13-plex pneumococcal dLIA platform developed by Pfizer, Inc., can generate 143 tests on a single assay plate, while the ELISA platform would require 39 assay plates and a great deal more liquid consumables to achieve the same number of tests. Also, there are obvious gains in efficiency, as the laboratory personnel can process more samples for the dLIA platform in the same amount of time.

**FIG 5:**
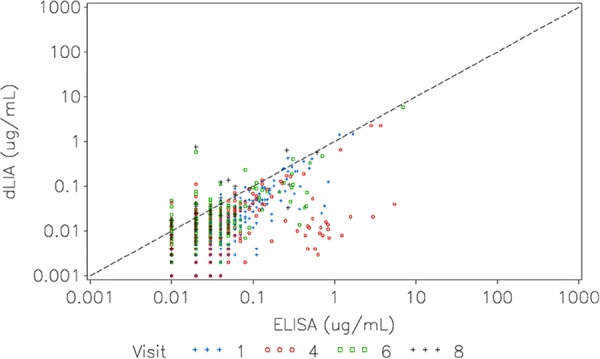
Scatter plot of the relationship between pneumococcal dLIA and ELISA IgG results in serum samples from prevaccinated infants for pneumococcal serotype 1. The scatter plot shows lower IgG measurements in prevaccinated infants and therefore greater specificity by dLIA than by the ELISA platform. Similar results were observed for the other serotypes.

One of the most important improvements provided by the dLIA platform over the ELISA is the enhanced specificity of IgG measurements, which is apparent in serum IgG measurements from unvaccinated subjects. This feature of the dLIA platform might lead to improved correlation between IgG and OPA assay measurements in adult clinical studies. Note that some level of IgG cross-reactivity was observed between the structurally related serotypes, 6A and 6B, as well as between 19A and 19F, but no interference was observed in the multiplex assay (18). Also, the dLIA has a greater dynamic range than the ELISA. Figure 5 shows the direct relationship between dLIA results and ELISA results on samples from unvaccinated infants (prior to vaccination) for serotype 1. Note the higher IgG results indicated by ELISA than by the dLIA platform; a similar pattern was observed for the other 12 serotypes examined in this study (data not shown). This outcome provides an explanation for the observed leftward shift of the dLIA RCDC and the greater separation between IgG measurements from vaccinated and unvaccinated subjects in matched serum samples. On the basis of these observations, it is expected that IgG fold-rise estimates by the dLIA platform would be higher than the ELISA estimates for all serotypes.

The ELISA consistently measured higher levels of IgG antibodies than the dLIA platform at the low end of the assay range. This observation was most apparent for serotypes 5, 6B, and 19A in subjects immunized with 7vPnC, which does not include serotypes 5 and 19A. This finding necessitates reduction of the threshold values for the dLIA platform for serotypes 5 (0.23 µg/ml), 6B (0.10 µg/ml), and 19A (0.12 µg/ml) in order to maintain the proportion of vaccine responders observed by ELISA. The 0.35 µg/ml benchmark is an appropriate dLIA cutoff value for serotype 6A despite the moderate location shift of the Deming regression line relative to the scale. This result is largely due to the fact that the regression curve crosses the theoretical *y* = *x* line of concordance near 0.35. For serotypes 1, 3, 4, 7F, 9V, 14, 18C, 19F, and 23F, the 0.35 µg/ml benchmark was shown to be a well-justified dLIA cutoff value.

Advances in the newer immunoassay methodologies have led to improvements in assay sensitivity and specificity and dynamic range, as well as to changes in IgG measurements compared to the older ELISA platform. A careful assessment of the dLIA platform developed by Pfizer, Inc., against the WHO ELISA using clinical samples from completed clinical vaccine studies has led to the selection of well-justified dLIA threshold values that preserve the percentage of vaccine responders observed in historical 13vPnC clinical trials. Our data support 0.35 µg/ml as the cutoff value for the dLIA platform developed by Pfizer, Inc., for serotypes 1, 3, 4, 7F, 9V, 14, 18C, 19F, and 23F. Lower threshold values should be used for serotypes 5 (0.23 µg/ml), 6B (0.10 µg/ml), and 19A (0.12 µg/ml) in order to maintain the proportion of vaccine responders that were observed by ELISA in completed clinical studies. This report provides well-justified threshold IgG concentrations for the dLIA platform developed by Pfizer, Inc., that correspond to the 0.35 µg/ml benchmark of the WHO reference ELISA platform.

## MATERIALS AND METHODS

### Serum samples.

A total of 1,574 archived serum samples were selected from 13vPnC and 7vPnC clinical trials conducted in infants and toddlers for reanalysis by the dLIA method based on the remaining sample volume and the serotype-specific IgG concentration by the WHO reference ELISA. Serum samples from four completed trials from the United States, Germany, and Japan were included (12, 14, 15, 19). Clinical studies 6096A1-006 and 6096A1-3024 were phase 3 noninferiority trials conducted in Germany and Japan, respectively (14, 19). Clinical study 6096A1-3005 was a phase 3 lot consistency trial conducted in the United States (15). These three trials supported Prevnar 13 (Pfizer, Inc.) registrations for infants. Serum samples from the post-primary-series immunization population and from the postbooster population were arranged into defined serum panels, as shown in Table 4. Note that preimmunization sera were not collected in these clinical trials. Therefore, paired pre- and postimmunization samples were obtained from study 6096A1-003, an early phase 1/2 trial in the United States, in which serum was collected prior to the first vaccine dose and again 1 month after the third immunization (see Table 4) (12).

### ELISA procedure.

The WHO reference ELISA was performed as described previously using the international reference standard serum, 89SF, to calculate all ELISA results (23, 24), which were reported previously (12, 14, 15, 19). The assays were carried out under double-absorbent assay conditions with pneumococcal absorbent (CWPS) and serotype 22F PnPS (CWPS2), as recommended by the WHO guidelines (7). Briefly, coated ELISA plates were washed with Tris-buffered saline containing 0.1% Brij-35 detergent. Serum dilutions were incubated in phosphate-buffered saline with 8 µg/ml of pneumococcal absorbent (CWPS) and 12.5 µg/ml of serotype 22F PnPS (CWPS2), and the reference standard serum, 89SF, was incubated under single-absorbent conditions with 8 µg/ml of pneumococcal absorbent without 22F PnPS. Serotype-specific IgG antibodies were detected with an alkaline phosphatase (AP)-conjugated anti-human IgG secondary antibody. The chromogenic AP substrate, p-nitrophenylphosphate, was dissolved in 1 M diethanolamine–0.5 mM magnesium chloride and added to reaction wells. Optical densities were measured on a spectrophotometer at 405 nm with a reference wavelength of 690 nm. Serum IgG antibody concentrations were calculated from the 89SF reference standard curve using log-log-linear regression.

### Multiplex dLIA procedure.

The dLIA procedure was performed as described elsewhere in this issue (18) using the international reference standard serum, 007sp, to calculate all serotype-specific IgG antibody concentrations.

### Bridging study objectives.

The primary goal of this study was to determine if the dLIA platform is a suitable replacement for the WHO reference ELISA. There were three main objectives. The first objective was to evaluate the linear relationship between the IgG antibody concentrations measured by the ELISA and dLIA. The second objective was to assess the performance of the multiplex dLIA platform in differentiation of pre- and postimmunization populations. The third objective was to provide a well-justified IgG concentration for each serotype assessed by the dLIA platform that corresponds to the 0.35 µg/ml efficacy threshold concentration established through the WHO ELISA.

### Statistical methods.

The primary endpoint for this bridging study was to derive serotype-specific serum IgG antibody concentrations, as determined by ELISA and multiplex dLIA platforms. The assay results were subjected to log transformation for analysis. All references to log transformation in this report correspond to the common logarithm with base 10. Statistical analyses were performed using SAS proprietary software version 9.2 (TS2M3).

A linear relationship was expected between the two platforms for postimmunization samples, since the two are similar in their underlying approaches. The methodology described by Tan and Iglewicz, also known as errors-in-variables regression or Deming regression, constitutes the basis for this analysis (16, 25). This methodology is strengthened when data are widely dispersed across the entire range. This methodology has been used in published work on similar assay platform comparisons (26). The precision ratio was set to a value of 1 for this analysis, and the best-fit linear regression line was used to find an alternative threshold value for each serotype by the dLIA method according to the established 0.35 µg/ml value by ELISA. Also, the cumulative distributions of IgG concentrations generated by both the ELISA and dLIA platforms were used to qualitatively visualize the separation between the pre- and postimmunization study populations for each serotype by reverse cumulative distribution curve (RCDC) (27).

The alternative efficacy threshold IgG concentrations for the dLIA were evaluated against the 0.35 µg/ml benchmark by calculating the proportion of vaccine responders in each study population according to serotype and threshold value. To compare the proportions of responders, two-by-two tables were produced for each of 14 serum panels, namely, A1, A2, A3, A4, B1, B2, B3, B4, C1, C2, C3, C4, D2, and D4 (see Table 1) for the seven 7vPnC serotypes, namely, 4, 6B, 9V, 14, 18C, 19F, and 23F. For the other six serotypes found in 13vPnC, 1, 3, 5, 6A, 7F, and 19A, two-by-two tables were produced for each of seven serum panels, A1, A2, B1, B2, C1, C2, and D2 (see Table 1). Data from these serum panels were combined to create a primary data set that included all assay data from all clinical study time points and a smaller secondary data set that included all assay data from only the post-primary-immunization time point.

These serum panels are representative of their corresponding underlying immunized populations at two key time points: 1 month after the infant immunization series (visit 4) and 1 month after the toddler dose (visit 6 or 8, depending on the trial). A two-by-two table was produced for each potential dLIA cutoff value and for each serotype and each serum panel.

For each two-by-two table, McNemar’s test was performed and its exact *P* value was used as an indicator of how well the proportion of responders by dLIA matched the proportion of responders by ELISA (20). The McNemar’s test was computed as *Q_M_* = (*n*_12_ − *n*_21_)^2^/(*n*_12_ + *n*_21_), where *n*_12_ and *n*_21_ were the counts of two mismatched cells (off-diagonal cells) in the two-by-two table. When both *n*_12_ and *n*_21_ are zero, McNemar’s test is not well defined. However, for our purpose, this scenario implies a perfect match between the ELISA and dLIA platforms. We use definitions of *Q_M_* = 0 and exact *P* value = 1 for such cases.

Fisher’s method was used to combine the 7 or 14 exact *P* values of the McNemar’s test for each alternative threshold value (21). For each serotype, if the combined *P* value corresponding to the 0.35-µg/ml cutoff value is 0.25 or greater, it would indicate that the 0.35-µg/ml cutoff value should be maintained for the dLIA platform. However, if the combined *P* value corresponding to the established 0.35 µg/ml benchmark is ~0.05 or smaller but the combined *P* value corresponding to the data-derived cutoff value is ≥0.25, it would indicate that the data-derived cutoff value is better justified.
